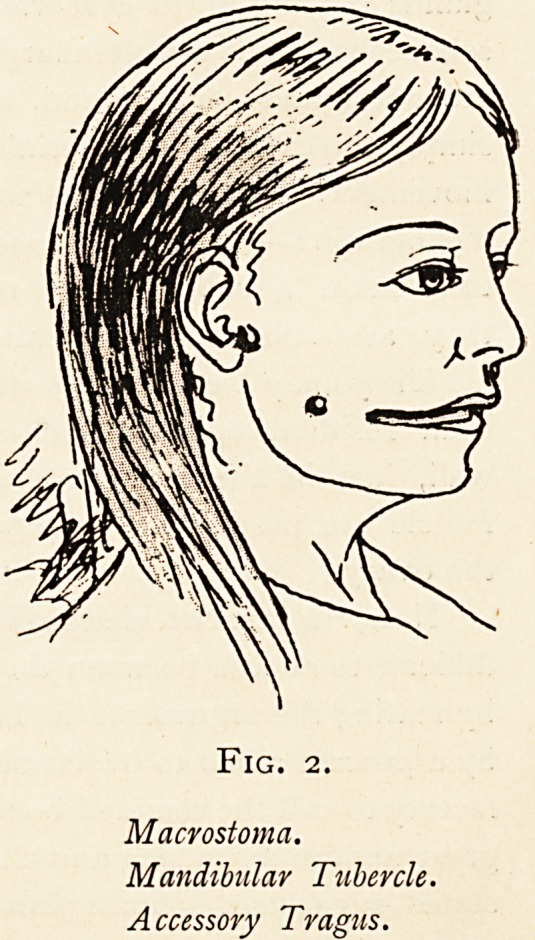# Dermoid Cyst of a Branchial Cleft

**Published:** 1896-06

**Authors:** Thomas Carwardine

**Affiliations:** House Surgeon, Bristol Royal Infirmary


### DERMOID CYST OF A BRANCHIAL CLEFT.

BY

THOMAS CARWARDINE, M.B., M.S. Lond., F.R.C.S. Eng.,

*House Surgeon, Bristol Royal Infirmary.*

A WOMAN with dermoid of the neck was admitted to the Bristol Royal Infirmary in February last, under the care of Mr. A. W. Prichard. She had a large bilateral tumour with a median isthmus in the submaxillary region, non-adherent to the skin and stationary on deglutition. The right lobe could be moulded, like putty, into any shape, and the fingers imparted their impressions to it. The left, harder and somewhat inflamed, had been incised four days before admission, when it is said fluid, like "fat," came away. Through the opening, the little finger could be inserted along the interior of the isthmus beyond the middle line. In addition there was a tubercle between the angle of the jaw and lobule of the ear, and there was a small nodule also upon the antitragus. (Fig. 1.)

The patient, who was 32 years of age, gave the following history: When she was 15 years old a small lump was noticed in the neck. A photograph taken two years afterwards shows a condition almost as bad as that on admission recently.

Her mother has a median hairy mole in the submaxillary region, and a small cervical tubercle about half-way down the anterior border of the left sterno-mastoid. The patient's children present no such abnormality.

Mr. Prichard incised the left side and dissected away a portion of the sac-wall as far as the tongue and body of lower jaw where it became adherent. The right side was similarly treated. The contents were removed by the finger, and consisted of about 5 ounces of a mass exactly resembling the stale, dry, hard roe of a fish. The elements of this were distinctly *soft*, were uniformly about the size of a millet seed, and to the naked eye a slight difference could be seen between the centre and periphery of each. Chemical examination showed the presence of albumen and globulin, urea to the extent of 0·2 per cent., phosphates and chlorides, and a trace of iron—probably from the presence of blood.

Microscopical examination of the small round bodies showed their epithelial structure and laminated arrangement, with an outer crust and central core. The cyst-wall exhibited the ordinary epithelial lining resembling skin with the superficial horny scales exfoliating. A supposed nodule in the cyst-wall led to the preparation of a section through the part. The nodule at first sight resembled a lymphoid nodule, but on examination with a high power it was seen that the cells composing the nodule were of epithelial and not of connective tissue type. This section is important; for if this nodule be the predecessor of one of the millet-seed bodies in the contents, it points to the origin of the roe-like material from epithelial cell-nests in the stroma of the cyst-wall.

It only remains to state the points of interest. The diagnosis and the treatment need no remark.

What strikes one at first sight, is that we are dealing with a dermoid condition of a branchial cleft, rather than a simple dermoid cyst. We have in this case proof that median dermoids of the neck may arise quite independently of the thyro-lingual duct. The isthmus of this tumour corresponds to such a median cervical dermoid.

Secondly, we have what we may call, for reasons presently to be explained, a branchial tubercle associated on the same side with an accessory antitragus. This demonstrates that branchial tubercles may arise independently of supernumerary or of cervical auricles.

Thirdly, this case affords evidence to show that the outer ear represents, not one operculum, but two opercula. The two sketches appended are for the comparison of Mr. Prichard's

FIG. 1.

*Branchial Dermoid.*
*Branchial Tubercle.*
*Accessory Antitragus.*

FIG. 2.

*Macrostoma.*
*Mandibular Tubercle.*
*Accessory Tragus.*

case with that of a similar condition occurring in the face. In Fig. 1, the case under consideration, an accessory antitragus is associated with a branchial tubercle, as we may call it for uniformity of nomenclature, and a dermoid of the 1st branchial cleft. In Fig. 2, we find existing together a supernumerary tragus (a common peculiarity), a mandibular tubercle on the cheek, and a macrostoma, all on the same side. Thus, in these two pathological examples we have clear evidence that the tragus represents the operculum related to the mandibular fissure, and the antitragus and its connections that related to the 1st branchial or hyomandibular cleft.

In the fourth place, I would draw attention to the peculiar contents of the tumour. Some four views suggest themselves as to their origin: (1) They may be epithelial pearls. But they are far more numerous than true pearls occur, and are soft instead of having the hardness of enamel. (2) They may be products of sebaceous glands. But there is no evidence of any sebaceous glands existing in the cyst-wall. Moreover, in ovarian dermoids sebaceous glands are often large and numerous, but such contents are not found—or if found, are very rare. (3) They may be simply the exfoliated epidermis rolled into shape by mechanical movement. The following note occurs in Mr. Bland Sutton's monograph:—"In rare cases dermoids have been found to contain a collection of rounded bodies resembling pills; they are collections of epithelial cells rolled into balls."[1] (4) They may be products of definite development, produced from residual epithelial cell-nests in the stroma of the cyst-wall, just in the same way as the elements of the Graafian follicle are produced from epithelial cell-nests in the stroma of the ovary.

Now, the last two views seem the only tenable ones, and it is difficult to choose between them. We may conclude, however, by stating the arguments in favour of the developmental view, by a process allied to ovulation in the ovary. It seems unsatisfactory to call the rounded bodies "pills" or "balls" when their great uniformity in size and character—so apparent in the fresh state—suggests a definite plan of construction by developmental agency. There is just as much reason theoretically for allowing that a hyomandibular dermoid should have roe in its contents, as that an ovarian dermoid should contain teeth. The fact that roe-like bodies like those in this case are not found in ovarian dermoids, or if so, very rarely, can be no argument against their occurrence in cervical dermoids; for it is characteristic of the contents of dermoids that they are *not* native to the part, and that there is usually an absence of structures normal to the part. For instance, in the case of ovarian dermoids, we commonly find teeth, hairs, horns, sebaceous glands, and so on, but not ova. Now, the genital ridge of epithelium from which the ovary is derived is essentially mucous membrane, and the ovarian follicles are essentially mucous glands. There is thus

[1] *Dermoids.* 1889, p. 8.

complete homology between the tissue of the cyst-wall and that of an ovary, for skin and mucous membrane are pathologically mutable and interchangeable. The epithelium of the genital ridge is a part of the epithelial lining of the primitive alimentary canal, for the cœlom or pleuro-peritoneal cavity was developed primarily by an abstriction from the primitive alimentary canal or archenteron.

The homology thus proved, what evidence have we that the conditions of development are analogous? We have first of all the microscopic appearances of the nodule in the cyst-wall. It is apparently composed of a mass of epithelial elements, somewhat encapsuled in the connective tissue: a nest of epiblast or hypoblast in a stroma of mesoblast. If this mass be the predecessor of one of the small bodies in the interior of such a cyst, it becomes at once obvious that they are not "masses of shed epithelium rolled into balls or pills."

To the naked eye, the resemblance, especially when fresh at the time of operation, to fishes' roe was very close. It is true that the rounded bodies are larger than human ova, but size is an element which has absolutely no morphological importance in the face of facts derived from structure and development.

The conditions of this tumour are comparable to those which obtain in the ovary of a frog in the breeding season, which consists of a main cavity with numerous diverticula lined with cells,—some, larger than the rest, being ova. The frog's ovary appears like a gland secreting ova into a main cavity.[1] It is unnecessary to prove structural identity in order to establish morphological homology. The arguments given are those in favour of the view that the roe-like rounded bodies are morphologically ova, using the term in a wide sense, homologous with the Graafian follicles of the ovary.

The alternative view is that they are simply masses of exfoliated epidermis rolled into balls or pills. The mechanical principles involved will commend themselves to all who have practised the art of dispensing.

[1] Bland Sutton. *Op. cit.*, p. 129.

## Figures and Tables

**Fig. 1. f1:**
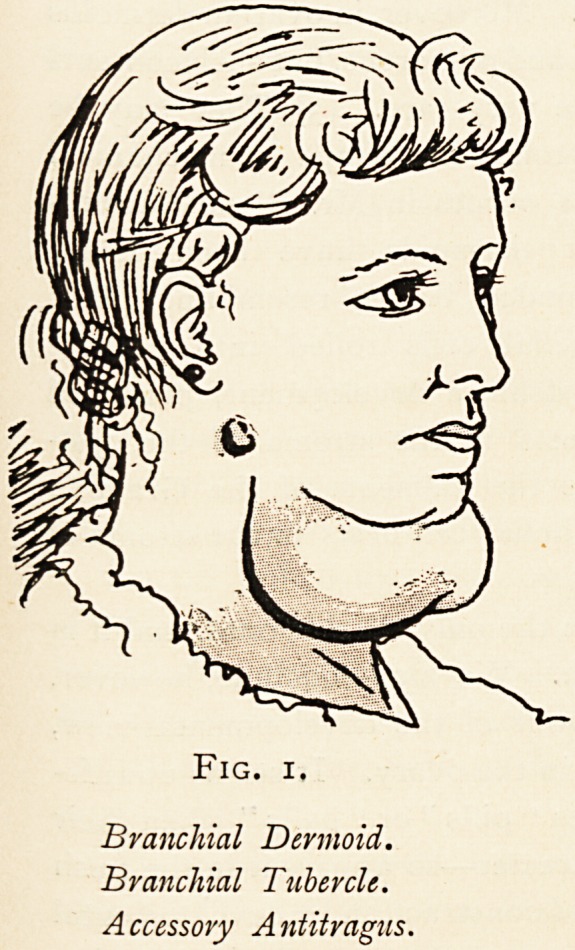


**Fig. 2. f2:**